# Modeling the relationship between maternal health and infant behavioral characteristics based on machine learning

**DOI:** 10.1371/journal.pone.0307332

**Published:** 2024-08-20

**Authors:** Zhiwen Yang, Xinyi Guo, Xuanzhi Chen, Jianfei Huang

**Affiliations:** School of Mathematical Sciences, Yangzhou University, Yangzhou, P.R. China; Hamadan University of Medical Sciences, School of Public Health, IRAN, ISLAMIC REPUBLIC OF

## Abstract

This study investigates the impact of maternal health on infant development by developing a mathematical model that delineates the relationship between maternal health indicators and infant behavioral characteristics and sleep quality. The main contributions of this study are as follows: (1) The use of Spearman’s correlation coefficient to conduct correlation analysis and explore the main factors that influence infant behavioral characteristics based on maternal indicators. (2) The development of a combined model using machine learning techniques, including random forest (RF) and multilayer perceptron (MLP) to establish the relationship between maternal health (physical and psychological health) and infant behavioral characteristics. The model is trained and validated by the real data respectively. (3) The use of the Fuzzy C-means (FCM) dynamic clustering model to classify infant sleep quality. An RF regression model is constructed to predict infant sleep quality using maternal indicators. This study is significant in gaining a deeper understanding of the relationship between maternal health indicators and infant development, and provides a basis for future intervention measures.

## Introduction

Infants are fragile and important individuals whose behavior is complex and influenced by many factors. The mother plays a vital role in the life of the baby, in addition to providing nutrition and physical protection [1], and it also gives the baby emotional support and security [2]. The mother’s participation in the child’s development is of great help [3]. These studies show that infant sleep and infant behavior have close relationship with maternal psychological characteristics.

Since infant sleep, infant behavior, maternal mental and physical characteristics are highly related and complex, there exist many works on exploring this relationship. The study of infant behavior can be analogous to the study of infant temperament. Infant temperament is influenced by many aspects, including maternal nursing behavior and early language, maternal and infant parenting pressure, and maternal and infant interaction [4]. In 2018, Prioreschi et al. reached a conclusion that the baby’s physical activity level was closely related to the mother’s activity level through studies [5]. The more closely the mother cares for the baby, the stronger the bond between the baby and the mother. In 2018, Klein et al. used cross-lag analysis to study the degree of bidirectional correlation between children’s temperament (fear, depression, positive influence, effort to control) and parenting behavior (warmth, negativity, limit setting, scaffolding, reactivity) [6]. Results showed that temperament and parenting have independent and additive effects on preschool-age child adjustment, with some support for a bidirectional relation. In 2021, Davies et al. examined the relationship between predictors (mother-infant relationship), outcomes (infant temperament), and mediators (maternal mental health, anxiety, and/or depression) through correlation analysis and concluded that mother-infant relationship had a significant indirect effect on infant temperament through anxiety [7].

Research on infant sleep is also important, and factors about early infant sleep are so complex, including child, parent and environmental factors. In 2016, Hairston’s study found that infant sleep patterns contribute to maternal mood, maternal sleep quality, perception of infant temperament, and maternal experience of intimacy [8]. In 2020, Geeraerts et al. examined associations between infant fussing and crying and self-regulation in early childhood and pre-school age, and concluded that the relationship between infant fussing and preschool self-regulation was inverted U-shaped, but only for children of highly sensitive mothers [9]. For babies whose mothers are less sensitive, fussing has nothing to do with later self-regulation. In 2021, Dias et al. found that maternal prenatal depression symptoms were associated with infant sleep problems, and infant sleep problems were associated with maternal postpartum depression symptoms [10].

At the same time, researchers had found that a mother’s favorable psychological and behavioral characteristics are also very beneficial to infant development, and parents and their parenting style play a key role in the development of self-regulation in young children, focusing on infancy, early childhood/preschool years, and early school years [11]. In 2012, Kim et al. found that infants were less self-regulated in non-reactive relationships (low parent-child interactivity orientation) but more self-regulated in reactive relationships (high parent-child interactivity orientation) [12]. In 2022, An et al. illustrated the multifinality of parent-infant processes and characterized their early relationship dynamics by examining observations and reports of parent-related (reactivity, resentment towards children, power claims, and invasiveness) and child-related (social-emotional competence, opposition, and anger) structures [13]. In 2023, Li et al. selected 108 Canadian postpartum women as samples and used multiple linear regression to examine the correlation between maternal and infant attachment and other variables, and found the importance of addressing maternal depression and maternal and infant attachment problems, which reduced the risk of childhood psychopathology [14].

While maternal influence on infant development is well-documented, the specific mechanisms remain unclear. This study aims to clarify the relationship by examining the interplay between maternal physical and mental health and infant behavioral patterns. EPDS(Edinburgh Postnatal Depression Scale) scores [15], HADS (Hospital Anxiety and Depression Scale) scores [16], CBTS (Childbirth Related Post-Traumatic Stress Disorder (CB-PTSD) Scale) [17]. This tool is widely utilized in clinical settings to assess the stress levels in women during the perinatal period. In 2012, Matthey et al. concluded using the EPDS and HADS score scales that about half of pregnant women had high EPDS and HADS scores on the first measurement [18], but that scores began to decrease after two weeks. In 2015, Thombs et al. used 10-item EBDS scores for perinatal depression screening, with the primary objective of determining the diagnostic accuracy of EPDS in detecting major depressive disorder in pregnant and postpartum women among all potentially relevant cut-off scores, and to consider patient factors that may affect accuracy [19].

Building on previous research that highlights the mother’s role in infant development, our study employs a novel approach to understand the nuanced relationship between maternal health and infant behavior. Random forest (RF) is a popular machine learning algorithm [20, 21]. It has many applications, can be used as prediction, classification and so on. RF can be combined or optimized for higher classification and prediction accuracy [22]. In 2023, Bicego et al. proposed a new RF clustering method, i.e., heterogeneous RF clustering, which is a novel unsupervised RF variant [23].

Multi-layer perceptron (MLP) is a popular machine learning algorithm [24–26]. In 2022, Said used multi-layer sensing machine artificial neural networks (MLP-ANN) to map and predict the thermal properties of multi-walled carbon nanotube nanofluids on the tube side and water on the shell side [27]. At the same time, through the *R*^2^ value of shell side model and the Kling-Gupta efficiency of tube side and shell side, it is concluded that the MLP-ANN model is not only robust, but also an effective prognostic model.

Fuzzy C-means (FCM) is a well-known and widely used as fuzzy clustering technique [28]. In 2022, Hu et al. first proposed a new FCM clustering algorithm, while continuing to compare five well-known FCM algorithms, and found that adaptive weight hybrid AFCM can enhance the performance of FCM more effectively [29].

RF algorithm is not sensitive to real data, but its excellent performance in feature selection can make up for the shortcomings of MLP. In 2022, Zou et al. used the integrated model of RF-MLP to estimate the ecological security level of cultivated land in the study area from 2019 to 2028 [30]. The model combined with MLP and RF model has high accuracy and is expected to achieve higher generalization and robustness in the classification prediction of maternal physical and mental health and infant sleep quality.

While previous studies have only used techniques such as simple linear regression or cross analysis to study the effects of maternal physical and mental health and infant behavioral characteristics, in order to study the relationship more deeply, our study used a combination (RF and MLP) of machine learning methods to further investigate the effects of mothers on their babies.

The work is organized as follows. In the section “Materials and methods”, we processed the data and established a relationship model between mothers and infants, combining MLP and RF models. Additionally, our proposed model was compared with other models in terms of AUC. In the section “Results”, the Spearman correlation coefficient was calculated for indicators between mother and infant, and the magnitude of the feature importance between maternal psychosomatic indicators and infant behavioral characteristics and sleep quality was also calculated. Finally, in the “Discussion” section, our conclusions were discussed with the results of previous studies, and in the “Conclusion” section, we have presented the conclusions and suggest that the relationship between the physical and mental health of mothers and infant development is important and provides a solid basis for future interventions, while also mentioning the limitations of the study in the “Limitations” section.

## Materials and methods

### Research framework

**Fig 1:**
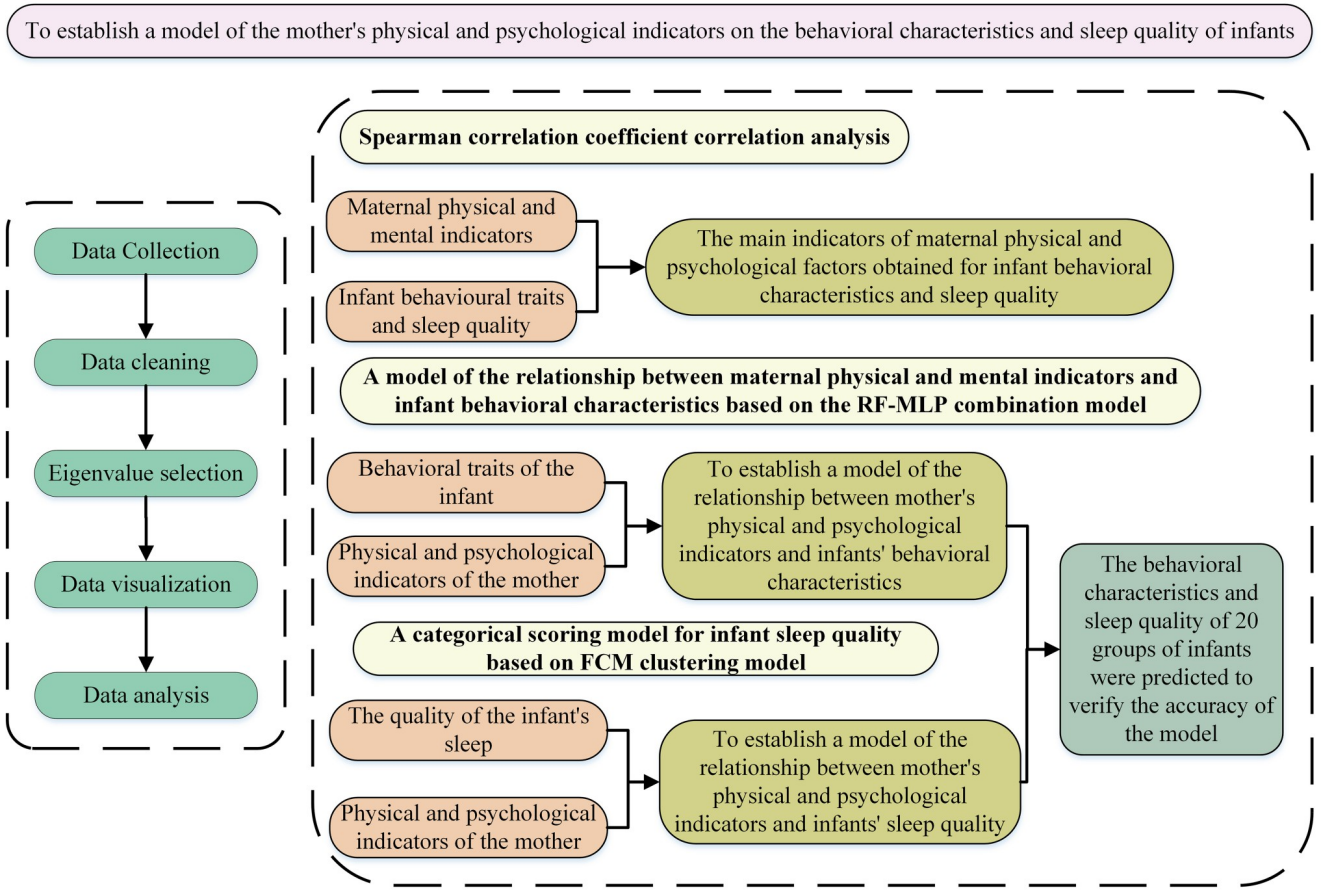
The framework of this study.

The study employs a combined model of RF and MLP to establish the relationship between maternal health (physical and psychological health) and infant behavioral characteristics and infant characteristics, and performs feature selection after data processing to simplify the model variables. According to the simplified variables, the relationship model between the infant’s behavioral characteristics and the mother’s physical and psychological indicators and the relationship model between the infant’s sleep quality and the maternal physical and psychological indicators are established. The accuracy of the model was verified by the known data, and 20 groups of infant data were finally predicted. Fig 1 shows the framework of this study.

### Data collection

The data derived from “Big Data and Mathematical Modeling Committee of the Chinese Society for Future Studies (CSFS)”(https://www.csfs.org.cn/) and “Tianjin Society for Future and Prediction Science”, a total of a number of data sets, including 410 3-to-12-months infant and its maternal health-related data. The data set collects a total of 410 groups of maternal physical and mental indicators and infant data, including maternal age, marital status, education level, gestation period, delivery mode, CBTS (the higher the score, the more obvious the symptoms. Symptoms of CB-PTSD including recurrent memories of the birth process, avoidance of birth-related stimuli, emotional numbness, irritability, insomnia, etc. can have a serious impact on a woman’s physical and mental health and social life), EPDS scores (short for the Edinburgh Postnatal Depression Scale, this scale is designed to help assess the severity of postpartum depression symptoms, including common symptoms such as low mood, insomnia, and changes in appetite, with higher scores indicating more severe symptoms), HADS scores (short for Hospital Anxiety and Depression Scale, this scale is used to assess whether a patient has symptoms of anxiety and depression while in the hospital, with higher scores indicating more severe symptoms) and the data of the infant’s behavioral characteristics, such as gender, age, sleep time throughout the night, wake times, and sleep mode. In the data, the infant behavior questionnaire, which is a scale used to evaluate the infant’s behavioral characteristics, emotions and reactions, is used to classify the infant’s behavioral characteristics into three types: quiet, moderate and contradictory. Among them, 1–390 groups of data contain all the indicators of mathor and infants, while 391–410 groups of data only give the physical and mental mathor’s indicators, and it is necessary to predict the behavioral characteristics and sleep quality of their infants according to the model. Table 1 shows the specific classification and numerical representation of each indicator in the data set.

**Table 1:** Data set indicator classification description.

	Indicator	Classification description

Mother	Age	/
	Marital status	unmarried(0), married(1)
	Education level	elementary school(0), middle school(1), high school(2), college(3), graduate school(4)
	Gestation period	gestational weeks
	Delivery mode	natural delivery(0), cesarean section(1)
	CBTS score	the range is 0–30, with a higher score indicating more severe symptoms
	EPDS score	
	HADS score	
Infant	Behavioral characteristics	quiet(0), moderate(1), contradictory(2)
	Gender	male(0), female(1)
	Age	/
	Sleep time	/
	Wake times	/
	Sleep mode	coax method(0), touch method(1), pacifier method(2), environment construction method(3), timing method(4)

### Data processing

**Fig 2:**
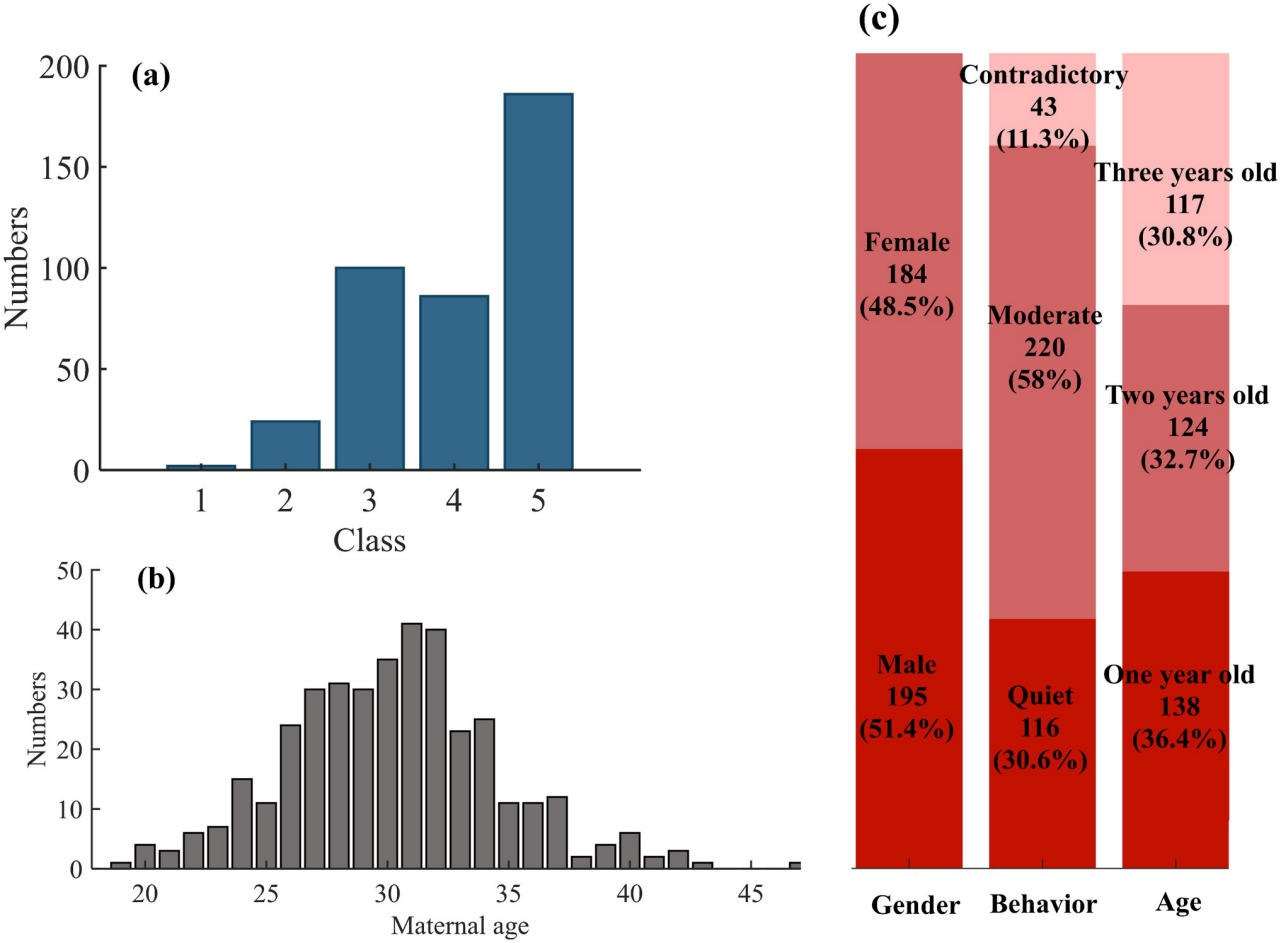
Statistical graph of categorical variables in data analysis. (a) represents the mother’s education level divided into five categories, as specified in Table 1. (b) is a distribution graph of the mother’s age. (c) shows the indicators of the infants, including the distribution of infant gender, behavioral characteristics, and age.

The given data set is preprocessed to remove the outliers. After removing abnormal data, 379 sets of valid data remained. Descriptive statistics and statistical images are performed on quantitative data such as maternal age, gestation period, and infants’ sleep time, as shown in Table 2 and Fig 2. Then, Spearman’s correlation coefficient method is used to explore the influence between mother’s indicators and infant’s indicators. The correlation coefficient *r*_*s*_ between the two groups of indicators *X*_*i*_ and *Y*_*i*_ is calculated in the formula shown in Eq (1), where *d*_*i*_ is the grade difference between *X*_*i*_ and *Y*_*i*_ [31],

$$\begin{array}{c} r_{s}=1-\frac{6\sum_{i=1}^{n}d_{i}^{2}}{n(n^{2}-1)}. \end{array}$$ (1)

**Table 2:** Descriptive statistical tables.

statistical indicators	Mean value		Standard deviation	Skewness		Kurtosis	
index	statistics	SE	statistics	statistics	SE	statistics	SE
*Maternalage*	30.26	0.23	4.41	0.29	0.13	0.51	0.25
*Gestationperiod*	39.15	0.09	1.78	-1.29	0.13	4.96	0.25
*CBTS*	5.99	0.26	5.01	0.93	0.13	0.28	0.25
*EPDS*	9.12	0.35	6.77	0.72	0.12	-0.21	0.25
*HADS*	7.88	0.22	4.28	0.56	0.13	-0.16	0.25
*Infantage*	1.94	0.04	0.82	0.10	0.13	-1.50	0.25
*Sleeptime*	10.11	0.07	1.45	-0.92	0.13	1.00	0.25
*Waketimes*	1.47	0.08	1.62	1.72	0.13	4.32	0.25

The abbreviations of Table 2: SE—Standard Error.

### Relationship model

Infant behavioral characteristics are mainly divided into three categories, namely quiet, moderate and quiet, moderate and contradictory. Therefore, the RF-MLP combined model is chosen to explore the relationship model between mother’s physical and mental indicators and infant’s behavioral characteristics, and predicts infant’s behaviors based on this model by using mother’s physical and mental indicators.

#### Multilayer Perceptron (MLP)

MLP model is a kind of feedforward neural network based on single layer perceptron extension, which has advantages in solving complex nonlinear problems. In the MLP model, each sample has d input features, that is, various physical and mental indicators of the mother. The matrix *X* ∈ *P*^n*d^ represents *n* small-batch samples, that is, *n* sets of data. Take a hidden layer as an example, let the number of hidden units be *h*, *H* ∈ *P*^n*h^ represents the output of the hidden layer, with hidden layer weight *W*^(1)^ ∈ *P*^n*h^ and hidden layer bias *b*^(1)^ ∈ *P*^1*h^. Meanwhile, if the sample has q output features, there are output layer weight *W*^(2)^ ∈ *P*^h*q^ and output layer bias *b*^(2)^ ∈ *P*^1*q^ After the affine transformation of the hidden layer, the activation function is applied to each cell:

$$\begin{array}{c} \sigma \left(x\right)=sigmoid\left(x\right)=\frac{1}{1+e^{x}}. \end{array}$$ (2)

After processing, the output *O* ∈ *P*^n*q^ of the output layer can be obtained [22]:

$$\begin{array}{c} \left\{ \begin{array}{c} H=\sigma (XW^{\left(1\right)}+b^{\left(1\right)}),\\ O=\sigma (HW^{\left(2\right)}+b^{\left(2\right)}). \end{array}\right. \end{array}$$ (3)

In establishing the MLP model, the physical and mental indicators of the mother are used as the input of the model, and the behavioral characteristics of the infant are used as the output. Mean Absolute Error(MAE) and Coefficient of Determination(*R*^2^) are used as statistical criteria to evaluate the performance of the model [32, 33]. The standard is defined as follows:

$$\begin{array}{c} MAE=\frac{1}{N}\sum_{i=1}^{N}|(x_{i}-y_{i})|, \end{array}$$ (4)

$$\begin{array}{c} R^{2}=1-\frac{\sum_{i=1}^{N}{\left({\hat{y}}_{i}-y_{i}\right)}^{2}}{\sum_{i=1}^{N}{\left(y_{i}-\bar{y}\right)}^{2}}, \end{array}$$ (5)

where *y*_*i*_ represents the true index value, $\bar{y}$ represents the average value of the true index value, ${\hat{y}}_{i}$ represents the predicted value.

#### Random Forest (RF)

The RF regression model can be summarized mathematically as follows: given a data sample *X* and a prediction set *Y*, on this basis, a forest dependent on random variable *θ* is planted to form a tree predictor *h*(*x*, *θ*_*k*_). The output result of this predictor is the infant behavior characteristics. The RF predictor is obtained by taking the mean value of these trees about *k*. The mean square generalization error of any tree predictor *h*(*X*) is given by taking the independent sample set which follows the distribution of random variables *X* and *Y* as the training set [34].

$$\begin{array}{c} E_{X,Y}{\left(Y-h\left(X\right)\right)}^{2}. \end{array}$$ (6)

The RF regression function is:

$$\begin{array}{c} Y=E_{\theta }h\left(X,\theta \right). \end{array}$$ (7)

When using the above formula, the approximate formula *Y* = *av*_*k*_*h*(*X*, *θ*) is usually used instead for sufficiently large *k*. Error analysis at this point is as follows (PE* represents the average generalization error of the RF):

$$\begin{array}{c} PE^{*}\left(tree\right)=E_{\theta }E_{X,Y}{\left(Y-h\left(X,\theta \right)\right)}^{2}. \end{array}$$ (8)

MLP can learn RF-optimized features to obtain more accurate prediction data on maternal psychosomatic indicators and infant behavioral characteristics and sleep quality. RF, as a statistical learning theory, models the decision tree of each sample by Bootstrap resampling, extracts multiple samples from the original sample, and then makes prediction by combining multiple decision trees. Finally, the final prediction is obtained by the following formula:

$$\begin{array}{c} \Delta =\frac{MAE_{1}-MAE_{2}}{max\{MAE_{1},MAE_{2}\}}, \end{array}$$ (9)

where *MAE*_1_ represents the average absolute error value of the multi-layer perceptron, and *MAE*_2_ represents the average error value of the RF. If Δ ≤ 0.05, then we use the following formula:

$$\begin{array}{c} Q_{1}=\frac{MAE_{1}}{MAE_{1}+MAE_{2}}, \end{array}$$ (10)

$$\begin{array}{c} Q_{2}=\frac{MAE_{2}}{MAE_{1}+MAE_{2}}, \end{array}$$ (11)

$$\begin{array}{c} Y_{pre}=Y_{preMLP}*Q_{1}+Y_{preRF}*Q_{2}, \end{array}$$ (12)

where *Y*_*pre*_ represents the final forecast value, *Y*_*preMLP*_ represents the prediction results of MLP, *Y*_*preRF*_ represents the prediction results of RF.

If Δ ≥ 0.05, one model with a higher average absolute error is discarded and the results of the other model are used as the final result.

#### Model accuracy verification

**Fig 3:**
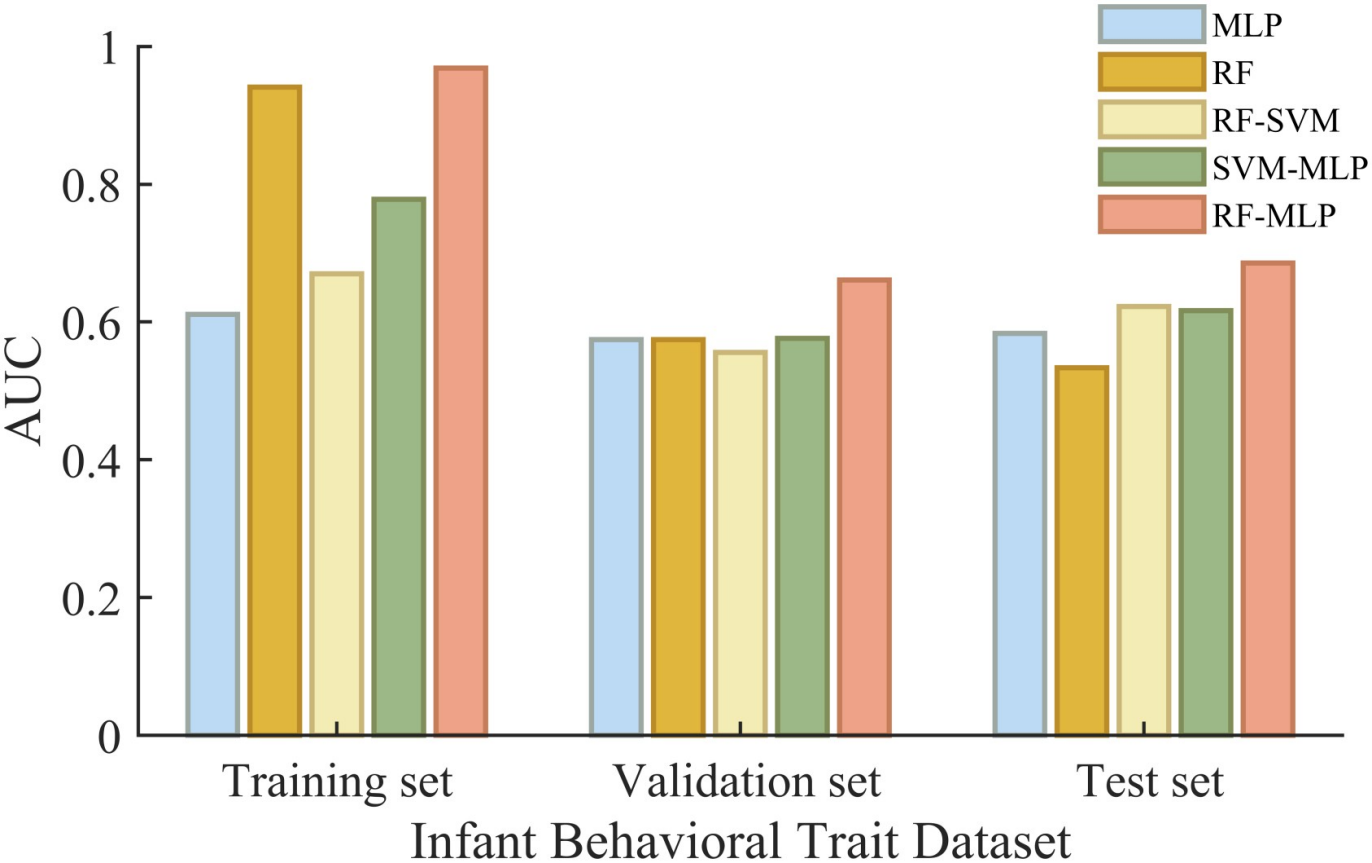
Comparing the proposed RF-MLP model with other models in the relationship between maternal indicators and infant behavioral characteristics. When comparing the performance of the models, the AUC (Area Under the Curve) is used as the evaluation metric [38]. To ensure a fair comparison, all models are trained, validated, and tested using the same proportions of datasets.

To validate the performance of the proposed RF-MLP model, psychosomatic indicators of the first 370 groups of mothers and data on infant behavioral characteristics and sleep quality are entered into the proposed model and other competing models. These competing models include MLP, RF-SVM (Support Vector Machine combined with RF), and SVM-MLP (Support Vector Machine combined with MLP), where SVM is a commonly used machine learning algorithm [35–37]. Finally, the accuracy (AUC) performance of MLP, RF-MLP, RF-SVM, and SVM-MLP algorithms is shown in Fig 3 and Table 3.

**Table 3:** The accuracy of the training, validation, and test sets of different methods.

Accuracy	Training set accuracy	Validation set accuracy	Test set accuracy
Method			
MLP	0.61	0.57	0.58
RF	0.94	0.57	0.53
RF-MLP	0.97	0.66	0.69
RF-SVM	0.67	0.56	0.62
SVM-MLP	0.78	0.58	0.62

Fig 3 and Table 3 show that the model combining the MLP method with RF or SVM is significantly better than MLP in terms of prediction, which is reflected in its higher AUC in the training, validation and test sets. Compared with the other two combined methods, RF-MLP has the highest prediction accuracy. The above results demonstrate the great effectiveness of RF in training, and also illustrate the progress and effectiveness of the RF-MLP model used in this paper in prediction.

### Classification model of infant sleep quality based on FCM clustering model

#### FCM clustering model

First of all, the data of the three indicators of sleep quality, that is, the whole night sleep time, the number of waking times and sleep mode, are normalized. In the Eq (13), *X*_*ij*_ represents the *j*-th value of the *i*-th indicator, *m*_*i*_ represents the minimum value of each indicator, *M*_*i*_ represents the maximum value of each indicator, and the normalized value *X*_*g*_ is obtained [29]:

$$\begin{array}{c} X_{g}=\frac{X_{ij}-m_{i}}{M_{i}-m_{i}}. \end{array}$$ (13)

Then, the FCM clustering model is established to classify the infant sleep quality into 4 categories. The model is to find the membership matrix *U* and cluster center *V* when the objective function *J* reaches the minimum value. Here, the least square error and criterion are selected as the clustering criterion of this question, and the clustering objective function is obtained as follows:

$$\begin{array}{c} J\left(U,V\right)=\sum_{i=1}^{c}\sum_{k=1}^{n}{\left(u_{ik}\right)}^{m}{\left(d_{ik}\right)}^{2}+\lambda \left(\sum_{i=1}^{c}u_{ik}-1\right), \end{array}$$ (14)

where the value of *J*(*U*, *V*) reflects the compactness within a class under some definition of difference. The smaller *J*(*U*, *V*) is, the tighter the cluster is, that is, the better the effect of the clustered class is. *u*_*ik*_ represents the membership degree of the *k*-th sample belonging to *i*-th class; *d*_*ik*_ represents the similarity between the sample point and the cluster center. Take the partial derivative of *u*_*ik*_, then get

$$\begin{array}{c} u_{ik}=\frac{1}{\sum_{j=1}^{c}{\left(\frac{d_{ik}}{d_{jk}}\right)}^{\frac{2}{m-1}}}. \end{array}$$ (15)

Using the same method, if *J*(*U*, *V*) is the minimum value, then the value of *v*_*i*_ is

$$\begin{array}{c} v_{i}=\frac{\sum_{k=1}^{n}\left(u_{ik}^{m}\right)x_{k}}{\sum_{k=1}^{n}\left(u_{ik}^{m}\right)},i=1,2,\ldots ,c. \end{array}$$ (16)

### Model of infant sleep quality based on maternal physical and mental

Based on the above classification of infant sleep quality, a RF regression model is established to predict infant sleep quality using maternal physical and mental indicators. RF, as a statistical learning theory, models the decision tree of each sample through Bootstrap resampling, extracts multiple samples from the original sample, and then makes predictions by combining multiple decision trees. The MAE and *R*^2^ used here are the same as in the previous section.

## Results

### Spearman correlation coefficient for correlation analysis

**Fig 4:**
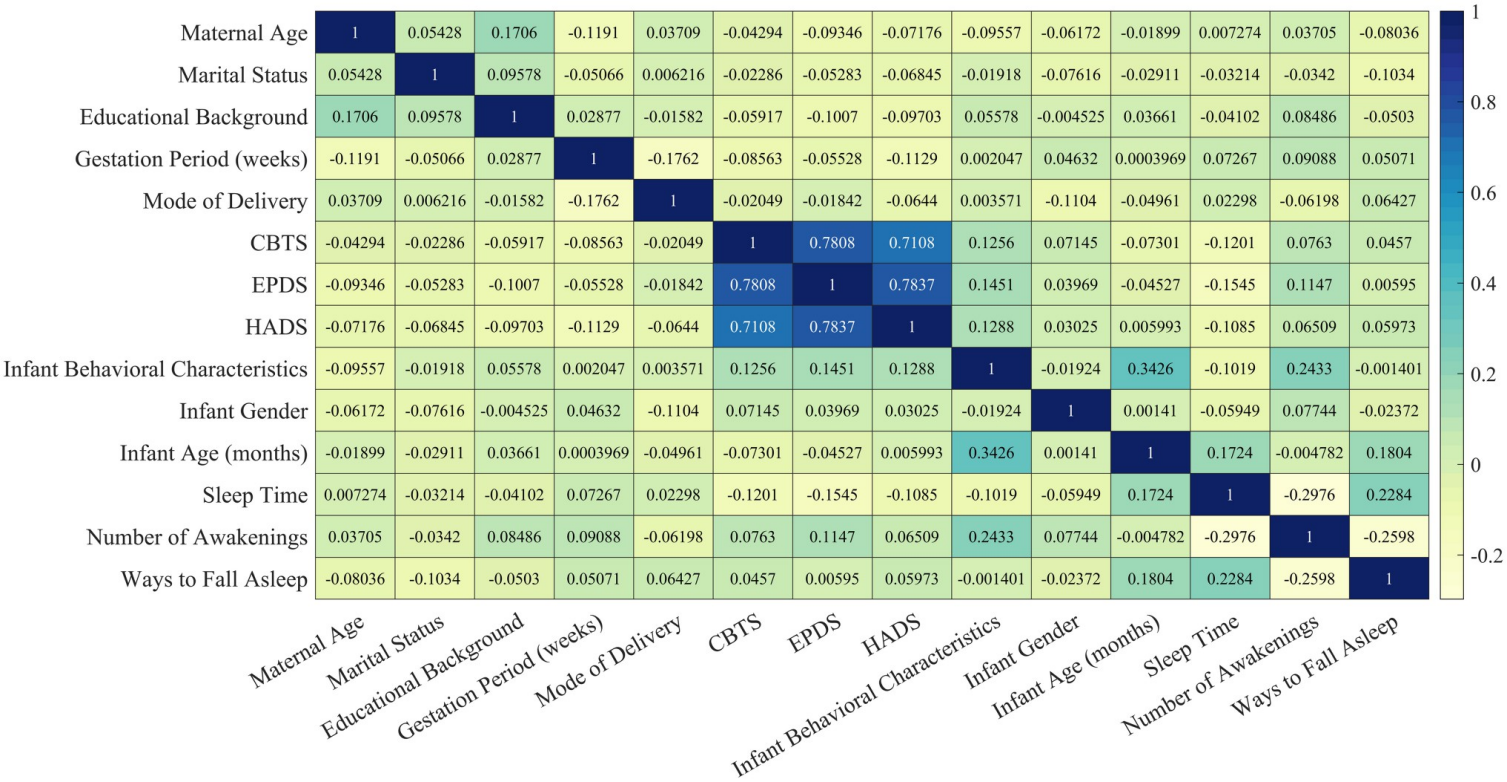
Spearman correlation coefficient heatmap. The Spearman correlation coefficient ranges from -1 to 1 and measures the strength and direction of the non-linear relationship between two variables.

Eq (1) is used to calculate the Spearman correlation coefficient to analyze the correlation between maternal physical indicators (age, marital status, education level, gestation period, and mode of delivery) and psychological indicators (CBTS, EPDS, HADS), as well as infant behavioral characteristics and sleep quality indicators (total sleep time, number of awakenings, and sleep onset method). Fourteen indicators are analyzed and the results are visualized as a Spearman correlation coefficient heatmap, as shown in Fig 4. The darker the color and the larger the correlation coefficient value, the stronger the degree of correlation. After organizing the data, the correlation between Maternal Age, Marital Status, Educational Background, Gestation Period (weeks), Mode of Delivery, CBTS, EPDS, HADS, Infant Behavioral Characteristics, Infant Gender, Infant Age (months), and Sleep Time can be analyzed. This analysis allows us to identify several factors with significant correlation coefficients.

The study primarily examines the relationship between maternal physical and psychological health and infant behavioral characteristics and sleep quality. Therefore, we will focus on the correlation between Maternal Age, Marital Status, Educational Background, Gestation Period (weeks), Mode of Delivery, CBTS, EPDS, HADS, Infant Behavioral Characteristics, Infant Gender, Infant Age (months), Sleep Time, Numbers of Awakenings, and Ways to Fall Asleep.

The analysis reveals that maternal age, gestation period, marital status, and mode of delivery have minimal influence on infant behavioral characteristics. However, maternal psychological indicators (CBTS, EPDS, HADS) significantly impact infant gender, behavioral characteristics, ways to fall asleep, and the number of awakenings. Moreover, as the values of these psychological indicators increase, indicating higher levels of maternal anxiety, infants tend to exhibit more contradictory behaviors and poorer sleep quality. Additionally, maternal educational background has a certain influence on infant behavioral characteristics, with higher levels of education associated with a higher prevalence of contradictory behaviors in infants.

In summary, based on the above analysis, we can conclude that certain maternal physical indicators (educational background and gestation period) have an impact on infant behavioral characteristics, with educational background having a stronger influence. Furthermore, specific maternal physical indicators (gestation period and mode of delivery) affect infant sleep quality, with the gestation period having a greater impact. Additionally, maternal psychological indicators (CBTS, EPDS, HADS) have an influence on both infant behavioral characteristics and sleep quality.

From Fig 4, it can be observed that several factors have relatively high Spearman correlation coefficients. These factors include: EPDS and Infant Behavioral Characteristics (0.1451), HADS and Infant Behavioral Characteristics (0.1288), CBTS and Infant Behavioral Characteristics (0.1256), EPDS and Numbers of Awakenings (0.1147), and so on.

### Feature importance and model performance of RF-MLP

**Fig 5:**
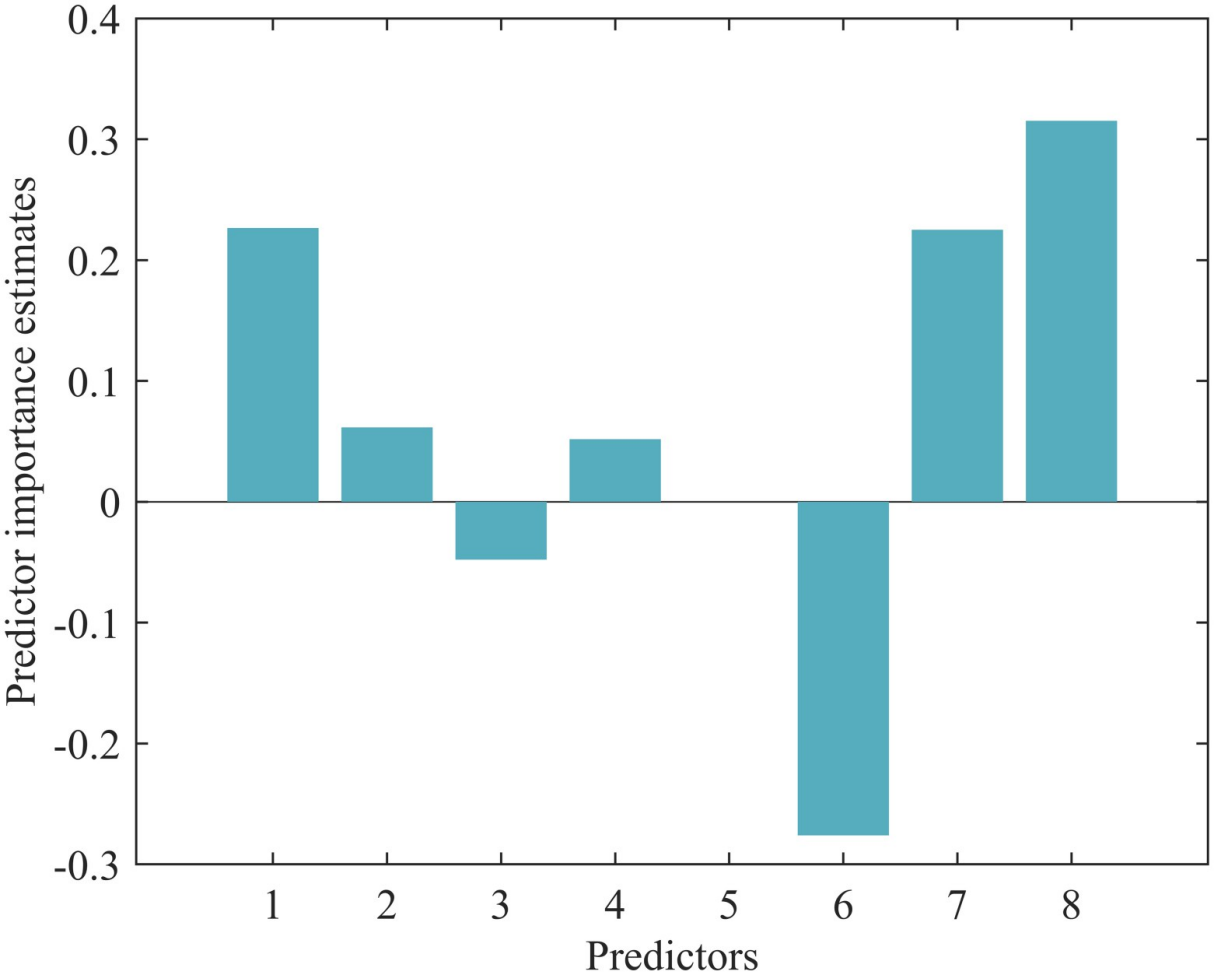
Feature importance. The feature importance values obtained from the tree model are generally relative rather than absolute. They indicate the relative importance of features in the model for predicting infant behavioral characteristics. Positive feature importance values indicate that the feature has a positive impact on infant behavioral characteristics, while negative feature importance values indicate a negative impact. The *x*-axis variables represent Maternal Age, Marital Status, Educational Background, Gestation Period (weeks), Mode of Delivery, CBTS, EPDS, and HADS.

Using a tree model to determine feature importance, we obtain Fig 5, which shows that HADS, maternal age, and EPDS have a significant impact on the target variable [39, 40]. They have high feature values in the tree model, indicating strong predictive power for the target variable and suggesting that they may be important predictors. Increasing maternal age may have a considerable influence on the target variable, implying that older mothers may have a greater impact on infant behavioral characteristics. CBTS also has a significant impact on the target variable, but its feature value is negative, indicating a negative correlation between CBTS and the target variable. In this model, an increase in CBTS may lead to a decrease in the target variable. The influences of marital status, gestation period, and educational background are relatively small, as indicated by their low feature values in the tree model, suggesting weaker predictive power and a lesser impact on the target variable. Based on the conclusions derived from the combination of Spearman correlation coefficients and the tree model, we find that the accuracy does not differ significantly when selecting 3, 4, or 5 features. Therefore, we select the variables Maternal Age, Educational Background, EPDS, and HADS.

**Fig 6:**
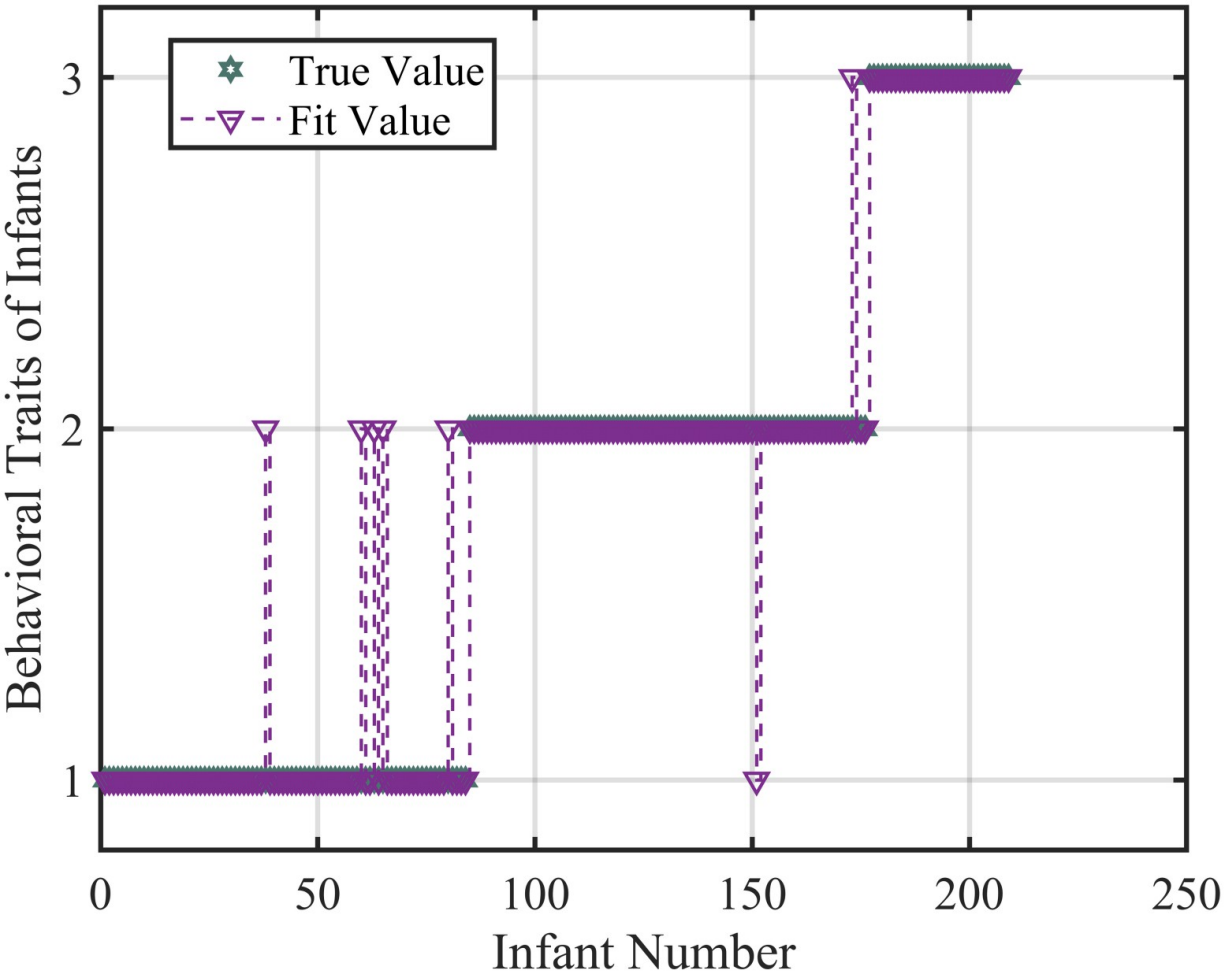
Training set fitting effect. Using Educational Background, CBTS, EPDS, HADS and Infant Behavioral Characteristics 370 groups of mothers and the known behavioral characteristics of infants, a RF-MLP classification model was established for prediction and a fitting plot was obtained. Among them, 1, 2, and 3 represent quiet, medium, and contradictory types, respectively.

To prevent overfitting, the model employed a random selection method, using 50 percent of the data as the training set, 20 percent as the validation set, and the remaining 30 percent as the test set. Based on the relationship model between infant behavioral characteristics and maternal physical and psychological indicators using the RF-MLP classification model, the training set’s fitting effect is shown in Fig 6. The RF-MLP model demonstrates high accuracy in predicting behavioral characteristics of infants based on maternal indicators. The confusion matrix of the trained model is shown in the figure, indicating that the prediction accuracy is above 94 percent, demonstrating excellent classification performance of the model.

**Fig 7:**
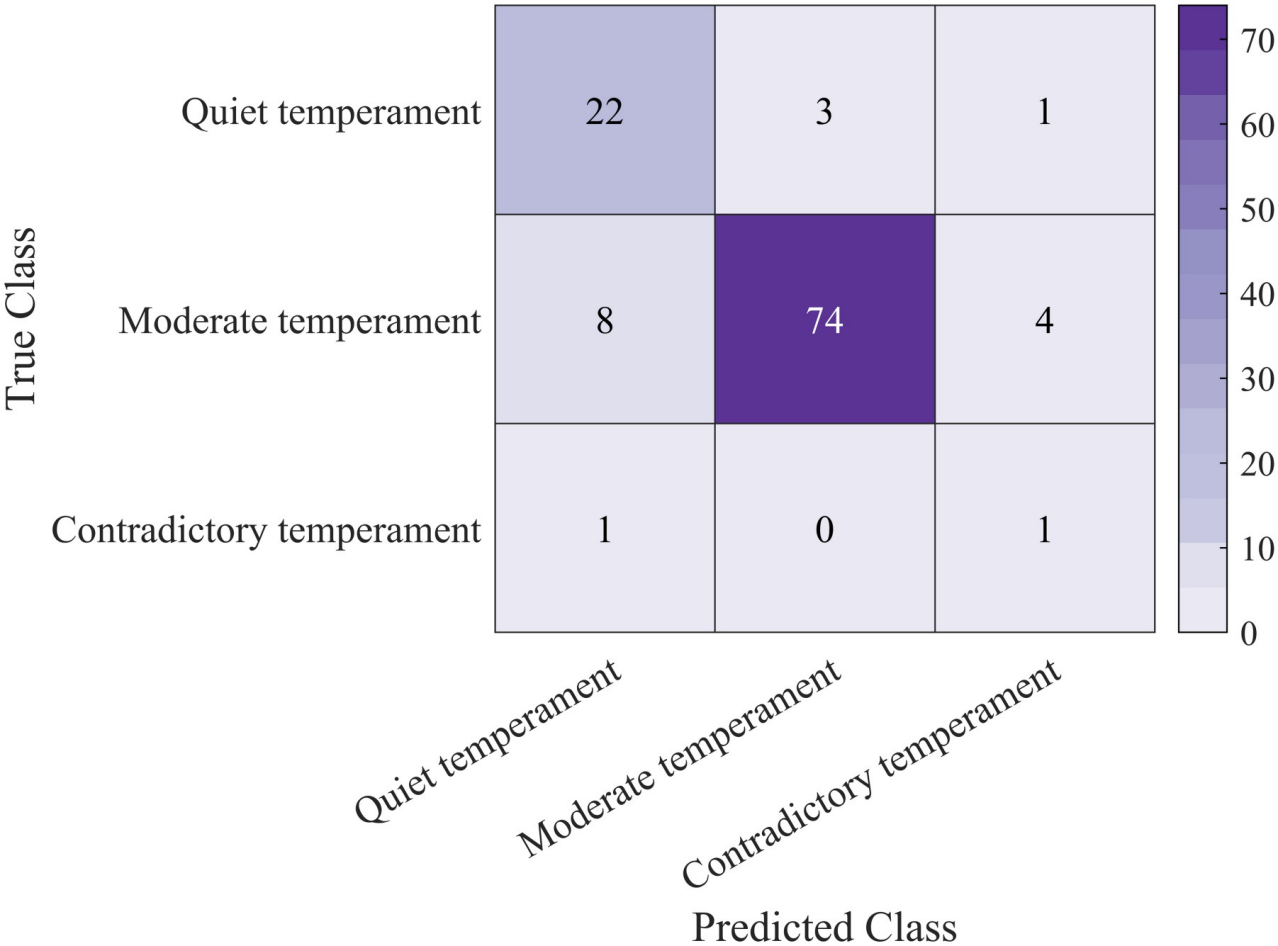
Confusion matrix.

The confusion matrix is used to calculate performance metrics by normalizing the counts of true positive and true negative predictions based on the sample size of the model. It displays the correct and incorrect classifications, as shown in Fig 7.

Using the RF-MLP classification model, we perform classification fitting with 370 sets of data, with 20 known data points used as unknowns. The validation results are shown in in Table A2 (see S1 File). In the table, Behavioral Features 1, 2, and 3 represent Quiet, Moderate, and Contradictory types, respectively.

Analyzing the results of predicting known trees using the RF-MLP model, we found that the model performed well on the given dataset. The accuracy reached 85 percent, meaning that the predicted values of 17 samples matched the true values exactly, with only 3 samples having different predicted values. This indicates that the model can accurately predict the categories of most samples. However, due to the small number of samples for Contradictory type infants, the predictions for this category are less accurate.

This study discovers the relationship between three infant characteristics and maternal physical and psychological indicators. For Quiet type infants, mothers had an age distribution between 25 and 36 years, higher levels of education, and lower scores in psychological indicators such as CBTS, EPDS, and HADS. For Moderate type infants, mothers had an age distribution between 23 and 31 years, moderate levels of education, and intermediate scores in psychological indicators such as CBTS, EPDS, and HADS. For Contradictory type infants, mothers had higher scores in psychological indicators such as CBTS, EPDS, and HADS, and their physical indicators included a higher age range of childbirth around 25 to 30 years and lower levels of education.

### The results of the classification scoring model

**Fig 8:**
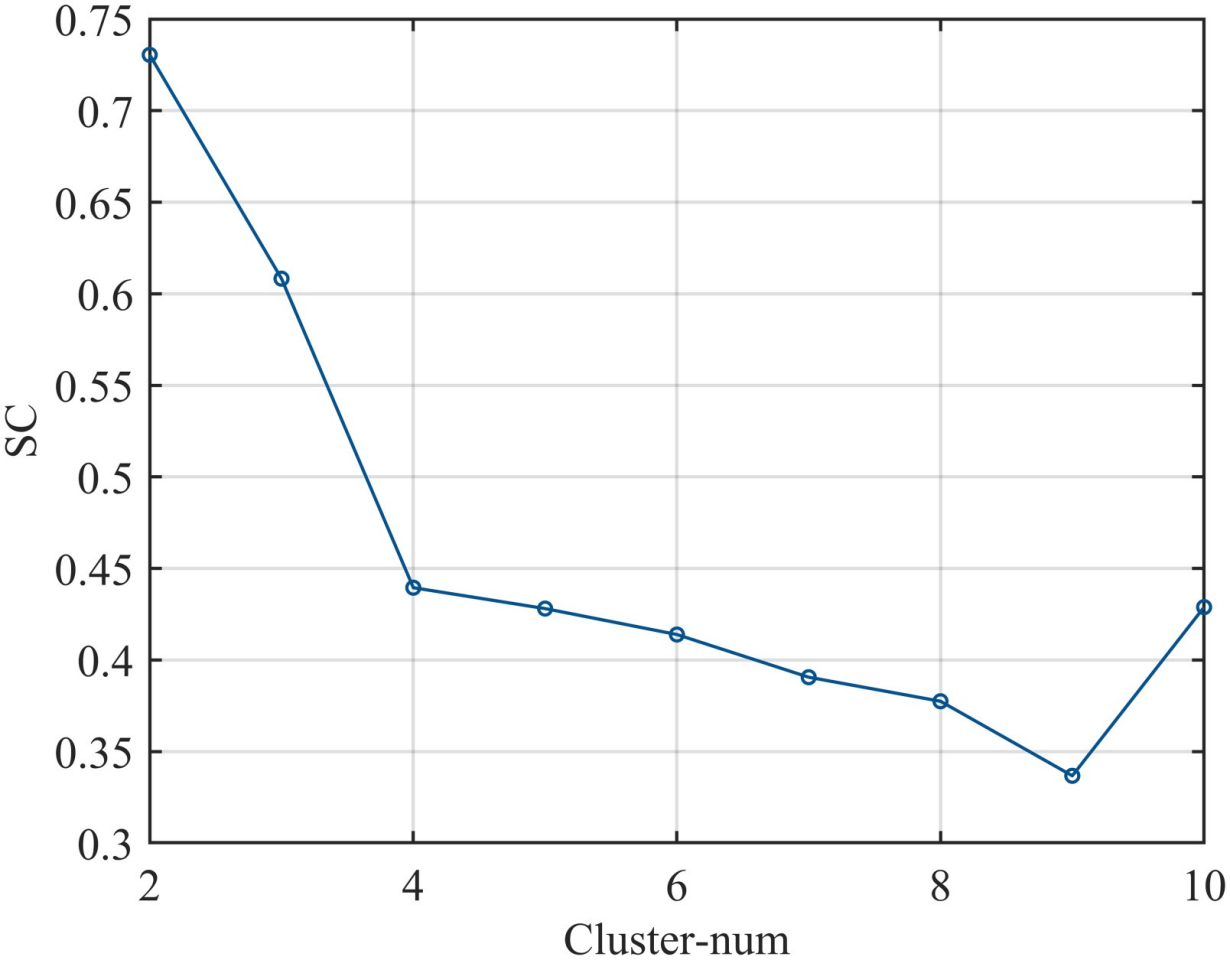
Silhouette coefficient curve.

**Fig 9:**
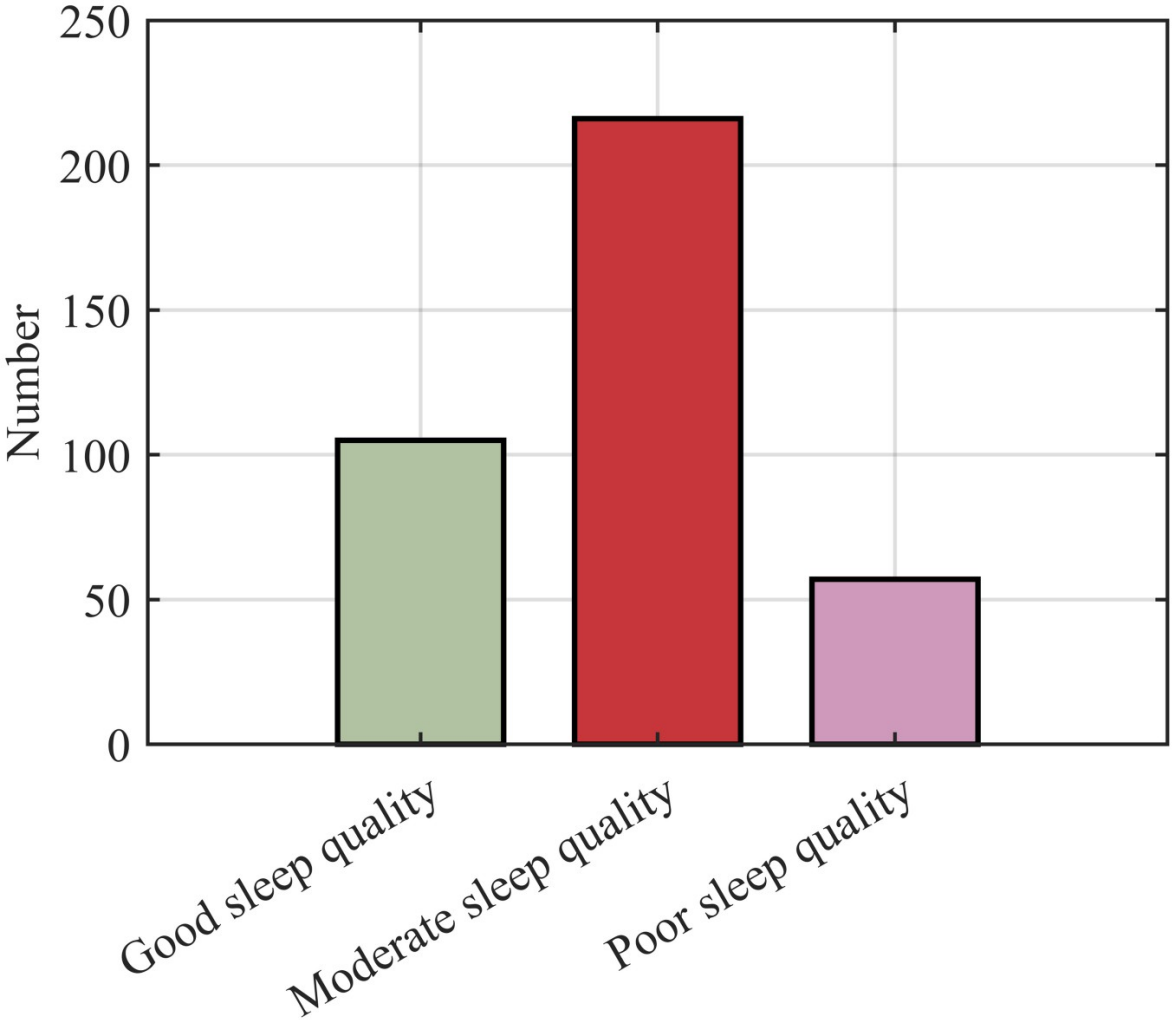
Statistical chart of sleep quality classification quantity. The numbers of good, moderate, and poor sleep quality were 105, 216, and 57, respectively, which were normally distributed.

The FCM algorithm is used to cluster and classify the sleep quality of infants based on factors such as total sleep time, number of awakenings, and sleep onset method. The clustering analysis employs the silhouette coefficient as a commonly used evaluation metric to measure the compactness and separation of the clustering results. A higher silhouette coefficient indicates better clustering quality. In the analysis process, we first plot the silhouette coefficient curve to assess the classification performance for different numbers of clusters [41], as shown in Fig 8. By observing the curve, we found that when the data was divided into three categories, the silhouette coefficient reached 0.61, indicating a good classification scenario. Subsequently, we implemented the FCM clustering algorithm using Matlab and obtained the clustering results for three categories, as presented in Fig 9. Considering both the silhouette coefficient and the clustering results, we can conclude that using the FCM algorithm to classify the sleep quality of infants into three categories yields satisfactory classification performance.

### Feature selection and validation results of infant sleep quality indicators

**Fig 10:**
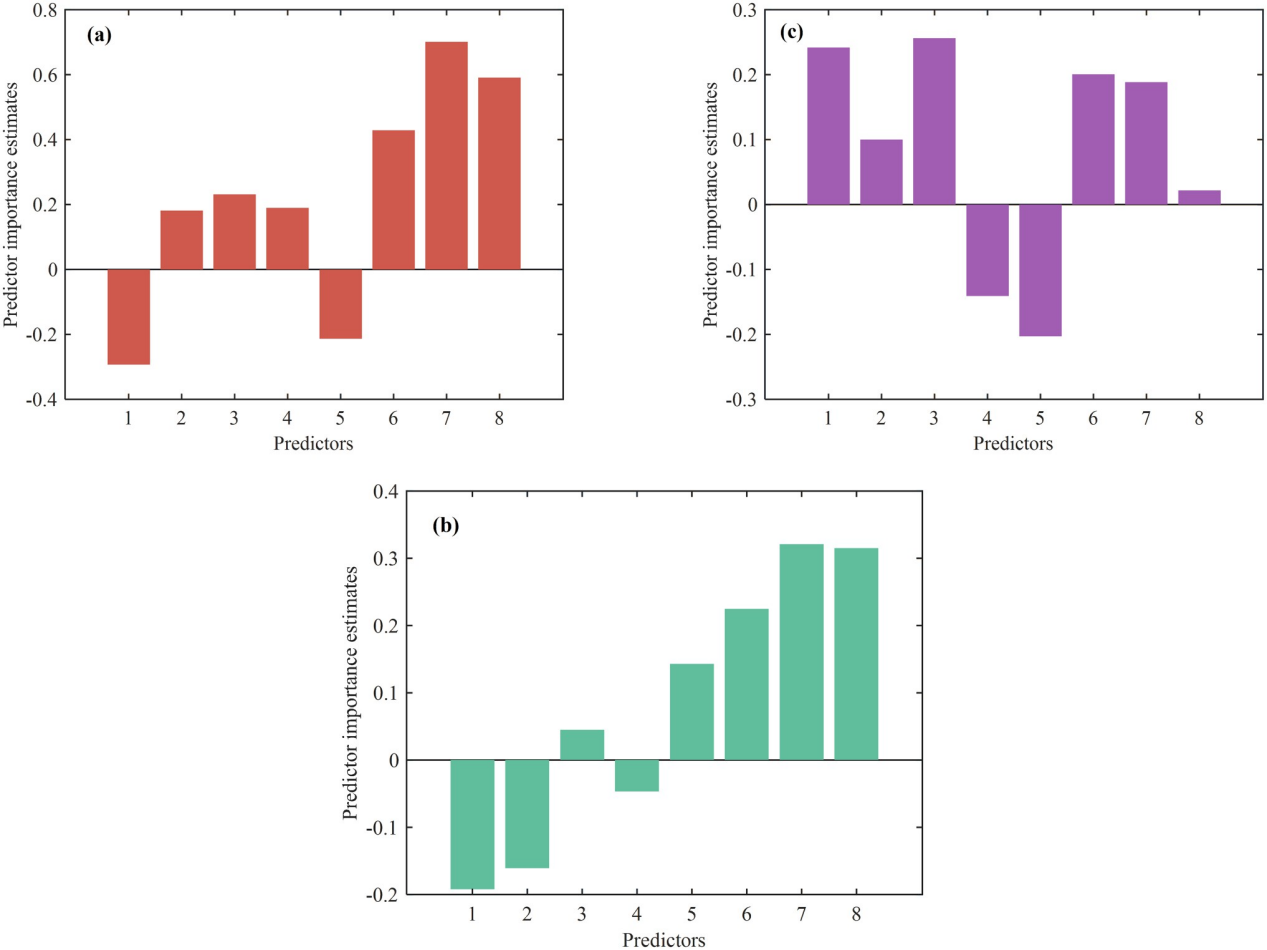
Feature importance. The *x*-axis variables represent Maternal Age, Marital Status, Educational Background, Gestation Period (weeks), Mode of Delivery, CBTS, EPDS, and HADS. Figs (a)–(c) represent the feature value distributions for the target variables of total sleep time, number of awakenings, and sleep onset method, respectively.

An RF classification model is used to model the sleep quality indicators of infants. To validate the model with known data, a tree model is used to determine the feature importance for the infant’s total sleep time, number of awakenings, and sleep onset method. The results are shown in Fig 10.

The study finds that EPDS, HADS, and CBTS had a significant impact on the target variable of infant sleep duration. In contrast, factors such as educational background, gestation period, marital status, mode of delivery, and maternal age had a relatively small influence. The accuracy of the results does not significantly differ when selecting 3, 4, or 5 features. Therefore, the chosen variables for the study are Maternal Age, Educational Background, EPDS, and HADS.

EPDS and HADS have a significant positive impact on the number of awakenings in the target variable. This suggests that mothers with higher levels of postpartum depression and anxiety may cause increased awakenings in their infants. The quality and patterns of an infant’s sleep can be affected by the emotional state of the mother. Additionally, trauma and stress experienced during childbirth may increase the number of awakenings in infants.

The number of awakenings in infants may be influenced by the physical and psychological condition of the mother during childbirth. The mode of delivery can also have a positive impact on the number of awakenings, as it may be related to the mother’s physical condition and the infant’s physiological adaptability. Maternal educational background and infant age had a relatively small impact on the number of awakenings, suggesting no significant effect. For the target variable of number of awakenings, we select EPDS, HADS, mode of delivery, and infant age based on the Spearman correlation coefficient.

The study finds that maternal age and educational background had a significant positive impact on the sleep onset method for infants. This indicates that mothers with higher education and older age may use more effective methods to help their infants fall asleep more easily. Other factors such as CBTS, EPDS, HADS, infant age, mode of delivery, and marital status also had some influence on the sleep onset method of infants.

To validate the known data, we use a set of 20 data points. The comparison between the actual and predicted values after handling missing data is shown in Table A2 (see S1 File). The predicted values align well with the actual values for most of the known data. The sleep onset method was evaluated by comparing the real data and validate data. The predictions for samples with IDs 370, 371, 372, 373, 376, 377, 379, 381, 382, 383, 385, 386, 387, 388, and 389 were accurate, resulting in an accuracy rate of 66.7 percengt. Similarly, for the sleep time throughout the night, the predictions for samples with IDs 371, 373, 374, 375, 376, 377, 378, 379, 381, 382, 383, 384, 385, 386, 387, 388, 389, and 390 were accurate, resulting in an accuracy rate of 85.7 percent. The accuracy rate for the Number of awakenings was 61.9 percent. Samples with IDs 371, 374, 376, 378, 379, 381, 382, 383, 384, 386, 387, 389, and 390 had accurate predictions.

## Discussion

The study has several implications for understanding the relationship between maternal physical and mental health and infant behavioral characteristics and sleep quality. We found that maternal psychological indicators and education level were associated with the increased incidence of contradictory babies, as well as the decrease in infant sleep quality. Our findings suggest that maternal psychological indicators and educational level are significantly associated with the incidence of contradictory infants and decreased infant sleep quality. This aligns with previous research by Denis et al. [42] and Dias et al. [10], which highlighted the link between maternal depression and infant sleep patterns. The use of the combined RF-MLP model has provided a convincing approach to modeling this complex relationship, with high accuracy on the training set, indicating a well-fitted model.

One of the main findings was the effect of certain physical indicators of the mother, such as gestation period and patterns, on the quality of the baby’s sleep. Specifically, we found that the gestation period has a significant influence, which corroborates Takegata’s findings [43]. This suggests that the gestation period may play a crucial role in shaping the sleep patterns of infants, a factor that could be further explored in future research.

However, our conclusion that maternal psychological indicators and education level are associated with an increase in contradictory infants and a decrease in infant sleep quality presents a contrasting view to Padilla’s research [44]. This discrepancy warrants further investigation and could be attributed to differences in study design, population, or cultural factors. It is essential for future studies to consider these variables and their potential interactions to gain a more nuanced understanding of the relationship between maternal health and infant development.

The significance of our study lies in its contribution to the body of knowledge regarding the intricate links between maternal physical and mental health and infant growth. By identifying specific factors that influence infant sleep quality and behavior, we provide a foundation for targeted interventions and support strategies that could enhance maternal and infant well-being.

## Limitations

However, there are also limitations in this paper. First, infants’ behavioral characteristics and sleep quality may be affected by many other factors, including certain maternal cortisol, tobacco, alcohol or certain illicit drugs, income factors, and maternal temperament traits, which can be important indicators for future research [43]. Secondly, the prediction results of MLP-RF are mainly based on the model’s performance on the training set, and practical applications may face more challenges and uncertainties. Thirdly, there are few data sets of people with low education in the data set of this paper, which may lead to the conclusion that mother‘s education is related to infant temperament, and it is contrast to Padilla’s findings [44]. Last, future studies should further incorporate more relevant variables, try different model structures, and consider multiple factors to more fully reveal the relationship between mother and infant.

## Conclusion

A new RF-MLP model was introduced to explore the intricate relationship between maternal physical and mental health and infant behavioral characteristics and sleep quality. Leveraging Spearman correlation coefficients, we analyzed the associations between a range of maternal indicators—spanning age, marital status, education level, gestation period, and delivery mode—and infant behavioral and sleep quality indicators. To classify infant behavior into quiet, medium, and contradictory types, we selected a support vector machine, which, given the dataset’s small size and high dimensionality, offered a balance between handling nonlinearity and avoiding overfitting.

Our model demonstrated high accuracy, with an AUC value calculated for the mother’s physical and mental health indicators and infant behavioral and sleep quality. The predictive model was tested on 20 groups of infants, revealing that most infants exhibited medium behavioral characteristics, with quiet types following and contradictory types being the least common. Sleep patterns were predominantly between 9 and 12 hours, with the majority of infants waking 0 to 2 times during the night.

The findings highlight the significant impact of maternal mental health and education on infant development, particularly the incidence of contradictory behaviors and sleep quality. These insights provide a solid foundation for designing future interventions and underscore the need for further research to encompass a broader range of variables and model structures. The study’s success in modeling and predicting infant outcomes based on maternal health metrics offers a promising avenue for advancing our understanding of early childhood development.

## Supporting information

S1 File Data of model. (DOCX)
