# Supplementary material for: Modeling the relationship between maternal health and infant behavioral characteristics based on machine learning
Source: PLoS One. 2024 Aug 20;19(8):e0307332. doi: 10.1371/journal.pone.0307332 (PMC11335109; doi:10.1371/journal.pone.0307332)
Supplement: S1 File — (DOCX) [file pone.0307332.s001.docx]

**Table A1. The Predicted Value from the Classification Model**

| IDs | Real Value | Validate data |
| --- | --- | --- |
| 1 | 2 | 2 |
| 2 | 1 | 1 |
| 3 | 1 | 1 |
| 4 | 3 | 1 |
| 5 | 2 | 2 |
| 6 | 1 | 1 |
| 7 | 1 | 1 |
| 8 | 1 | 1 |
| 9 | 1 | 1 |
| 10 | 2 | 2 |
| 11 | 1 | 2 |
| 12 | 2 | 2 |
| 13 | 2 | 2 |
| 14 | 2 | 2 |
| 15 | 2 | 2 |
| 16 | 2 | 2 |
| 17 | 2 | 2 |
| 18 | 1 | 2 |
| 19 | 1 | 1 |
| 20 | 2 | 2 |

**Table A2.** Comparing the actual value of the infant's sleep quality index with the predicted value predicted by the absence treatment

| IDs | Real data | | | Validate data | | |
| --- | --- | --- | --- | --- | --- | --- |
|  | Sleep onset method | Sleep time throughout the night | Number of awakenings | Sleep onset method | Sleep time throughout the night | Number of awakenings |
| 370 | 5 | 9 | 1 | 5 | 9 | 1 |
| 371 | 2 | 12 | 0 | 1 | 12 | 0 |
| 372 | 1 | 11 | 2 | 4 | 11 | 1 |
| 373 | 2 | 10 | 2 | 2 | 8 | 0 |
| 374 | 4 | 12 | 0 | 4 | 12 | 0 |
| 375 | 2 | 5 | 3 | 2 | 5 | 1 |
| 376 | 1 | 11 | 0 | 1 | 11 | 0 |
| 377 | 1 | 8 | 2 | 1 | 8 | 2 |
| 378 | 4 | 11 | 1 | 4 | 11 | 1 |
| 379 | 4 | 10 | 0 | 4 | 10 | 0 |
| 380 | 5 | 7 | 2 | 5 | 7 | 1 |
| 381 | 5 | 9 | 0 | 5 | 9 | 0 |
| 382 | 4 | 10 | 1 | 4 | 10 | 1 |
| 383 | 5 | 11 | 1 | 5 | 10 | 1 |
| 384 | 4 | 9 | 0 | 2 | 9 | 1 |
| 385 | 1 | 10 | 2 | 1 | 10 | 0 |
| 386 | 2 | 9 | 1 | 2 | 9 | 1 |
| 387 | 4 | 11 | 2 | 4 | 11 | 0 |
| 388 | 1 | 10 | 2 | 1 | 12 | 0 |
| 389 | 1 | 6 | 2 | 1 | 6 | 2 |
| 390 | 4 | 9 | 0 | 4 | 10 | 1 |
